# Did the cyberspace foster the entrepreneurship of women with children in rural China?

**DOI:** 10.3389/fpsyg.2022.1039108

**Published:** 2022-10-25

**Authors:** KaiChao Shao, Ruixue Ma, Lulu Zhao, Kai Wang, Joseph Kamber

**Affiliations:** ^1^Institute of Food Economics, Nanjing University of Finance and Economics, Nanjing, Jiangsu, China; ^2^School of Marxism, Xinjiang Normal University, Urumqi, Xinjiang, China; ^3^Graduate School of Management, Management and Science University, Shah Alam, Selangor, Malaysia; ^4^Department of Foreign Language Studies, Lingnan Normal University, Zhanjiang, Guangdong, China

**Keywords:** rural women with children, entrepreneurship, transmission mechanism, inclusive economic development, infotechnology

## Abstract

Female-entrepreneurship plays a significantly important role in rural areas of China today. In fact, it is a driving force behind inclusive economic development of the country as a whole. However, notably very little literature out there has focused on the impact of how widespread usage of information technology tools affects the mothers entrepreneurship in the outskirt regions. Here, in this paper, the authors attempt to explore the finer details of such an impact by utilizing the data from the 2017 *China Integrated Social Survey*; along with the *IV-Probit* model to explore the transmission mechanism. Interestingly enough, it was discovered that Internet applications and tools do indeed increase entrepreneurship among women with children by a roughly 7.88%. In addition, this paper finds that the utilization of such “*InfoTech*” promote a continuous progress in entrepreneurship among those women with children who endeavor to start a self-managed enterprise in the less developed areas. Lastly, when looking the analysis of the transmission mechanism, this paper found that the role of Internet-usage in promoting rural mothers entrepreneurship was mainly through three factors – the gender equality effect, the information learning effect, and the financing effect. This accumulated data will be thoroughly presented here in the ensuing sections.

## Introduction

Female entrepreneurship has been an important catalyst for inclusive economic development; thus, alleviating employment pressures, assuring women out of poverty (whilst simultaneously improving their status in society) and eliminating gender bias (Brixiová and Kangoye, 2016; Ojong et al., 2021). On the contrary, although an increasing number of female entrepreneurs have made significant contributions to economic growth over the last decades (Deng et al., 2011; Aggarwal and Johal, 2021), their numbers are still far less than males; especially observed in developing countries and rural areas; says (Hattab, 2012; Noguera et al., 2013). This is due to the several influential variances: family division of labor, house, chores, child raising …etc. (Hossain et al., 2009), cultural environment (Rosca et al., 2020), technological conditions of the said areas (Tan and Li, 2022), and character attributes (Marlow, 2020).

According to the white paper “*Gender Equality and Women’s Development in China*,” the proportion of female entrepreneurs rose from 20 to 25% between 2005 and 2015 while the proportion of rural single-women starting own businesses showed a low increase of only 7.6% (Wang and Lin, 2019); clearly surpassing the unnoticeable rise in the number of married and/or female parents enterprise in rural areas.

As a unique group, rural mothers’ participation in employment and entrepreneurial behaviour is strongly obstructed and challenged by marital obligations and duties; mainly, family responsibilities (Looze and Desai, 2020; Chu et al., 2021). When contrasting rural male entrepreneurs to rural women with children entrepreneurs, we see that the latter face more severe constraints in terms of household chore-obligations, gender perceptions and access to resources (Liu, 2013; Cooke and Xiao, 2021). And, when comparing our target group (mother entrepreneurs) with urban and rural unmarried-female entrepreneurs, we find that the rural mother holds the lowest educational attainment, cognitive level, and entrepreneurial environment, however, marriage and childbirth responsibilities are higher (Archer, 1996; Kurtege Sefer, 2020).

The disadvantageous position of rural mothers make their entrepreneurial conduct unique (Patrick et al., 2016). Many scholars refer to this phenomenon as ‘*Motherhood Penalties*’ (Budig et al., 2012; Kahn et al., 2014). This is partly due to the influence of traditional gender protocols on women, and partly due to the fact that rural entrepreneurship often faces more severe resource constraints (Ascher, 2012; Kavita, 2013; Sema and Kürşat, 2020).

As mentioned earlier, we found a rich body of literature on female entrepreneurship, but little to none on the entrepreneurial behaviour of rural married women raising children in China. It is estimated that there are roughly 135 million married and fertile women in rural China. Consequently, supporting these women’s entrepreneurial enthusiasm in order for them to play a more vital role as drivers of the rural economy has become an important part of China’s inclusive rural economic development (Si et al., 2022a).

Conversely, the other side of the coin reveals that the perpetual development of information technology has profoundly altered the human society in ways unimagined decades ago (Alderete, 2014). Consider how the Internet contributed a positive innovative effect on information gathering causing a boom in human capital with business transactions among other things (Caceres-Diaz et al., 2019). Ultimately, having adequate information is an elemntal factor in enabling individuals to identify entrepreneurial opportunities (Shane and Venkataraman, 2000). Hence, the Internet can surely provide rural residents with valuable information for their business ventures.

According to a survey by the *China Internet Network Information Centre* (CNNIC) and the *Ministry of Agriculture and Rural Development*, the number of Internet users in China peaked at 1.032 Billion in 2021 with access rate of no less than 73.0%. Meanwhile, the number of rural Internet users soared at 284 Million with accessing rate of 57.6%. Furthermore, 15.205 Million rural online shops (e-commerce) have been utilizing the World Wide Web to start their own businesses.

Addressing women in business, in May 2017, the *All-China Women’s Federation’s Opinions on Launching Women’s Action on Entrepreneurship and Innovation* proposed that women should be encouraged and assured to make use of the Internet in order to innovate and to start own businesses; thus playing a “half-hearted” role in mass movement of entrepreneurship and innovation. When reading such news, the question, “Did the Cyberspace Foster the Entrepreneurship of Women with Children in Rural China?” comes to mind. And, more specifically, in what way does this affect the entrepreneurship of pregnant women in rural areas? These two questions are the main focus addressed in this paper.

Based on the gathered data from *China General Social Survey* 2017 (CGSS), this paper attempts to validate the impact of Internet technology application on entrepreneurial activities of rural married-pregnant women. Moreover, it further explores the transmission mechanism between the two sides; namely, Internet technology usage and the married-pregnant women’s entrepreneurship in rural regions.

The marginal contributions of this paper include the following:

1. The analyzation of the entrepreneurship of married-women-with-children in rural areas from the perspective of Internet technology usage; which, in turn, broadens the research on women’s entrepreneurship in general. It will, also, put to attention important implications for promoting digital economy, rural revitalization and inclusive economic development.
2. The exploration of the aforementioned transmission mechanism from the angle of several factors – gender perceptions, learning effects and economic empowerment; thus, enriching the research content of female entrepreneurship.
3. A comparison between individual and environmental differences of the impact the InfoTech has on rural women’s entrepreneurship; this being from the perspective of individual characteristics and the heterogeneity of the entrepreneurial environment of rural married and pregnant women.

## Theoretical analysis and research hypothesis

Gender plays an important role in entrepreneurial behavior (Akehurst et al., 2012). Although female entrepreneurship has made great contributions to promoting high-quality (inclusive) economic development, there is a certain difference between male and female entrepreneurs, with the proportion of female entrepreneurship being much lower than that of males (Georgellis and Wall, 2005). This is not only related to gender differences and social culture, but also related to entrepreneurial motivation and family division of labor (Minniti and Nardone, 2007; Shastri and Pareek, 2019). Generally speaking, women’s entrepreneurial motivation is similar to men’s in many ways, but it also differs significantly (Minniti, 2010). On the one hand, the entrepreneurial environment of women is often influenced by social perceptions and cultural environment and facing more issues similar to gender stereotypes and gender discrimination, which makes it more difficult for women to start businesses. By using Gender and Enterprise Development in Africa survey data, Franzke et al. (2022) found that gender bias hinders women’s economic empowerment and widens the gender gap in entrepreneurship. Zhou et al. (2019) found that gender inequality significantly inhibits rural women’s entrepreneurial performance based on data from the Thousand Villages Survey in China.

On the other hand, there is the effect of household division of labor, married women with children bear more family responsibilities than men and single women, and they may face significant constraints in pursuing any roles other than housewives, thus, the goal of entrepreneurship among married women with children is often based on the balance between family and work (Nguyen et al., 2014). Kobeissi (2010), based on a study of female entrepreneurship data from the Global Entrepreneurship Monitor (GEM) report, found that in developing countries, women’s contributions are evaluated more from fertility and family perspective than from production and income perspective, and that cultural and social rules constraints affect female entrepreneurial behavior. DeMartino and Barbato (2003), using a sample of 1763 entrepreneurs in the United States, found that women consider entrepreneurship as a career choice more because the flexibility of self-employment enables them to balance their professional and family responsibilities. In conclusion, married women with children entrepreneurs face more constraints than male and unmarried female entrepreneurs.

Underdevelopment and the unique geographic, economic and social characteristics of rural areas may impose more constraints on married women with children entrepreneurship than in urban areas (Stathopoulou et al., 2004). First, the innovative entrepreneurial environment in rural areas may inhibit entrepreneurship among married women with children. Due to remote location, underdeveloped of commerce and lagging information, the lack of opportunities for entrepreneurship and employment in rural areas hinder rural married women with children entrepreneurship (Warren-Smith, 2012); second, social attitudes and family responsibilities have a great impact on rural married women with children. In such social environment and family division of labor, women not only need to devote more energy to parenting and supporting the elderly but also have to pay more time to household chores. This undoubtedly makes it more difficult for married women with children in rural areas to start their businesses. Finally, rural female entrepreneurs face serious financing constraints, due to the lack of financial institutions, insufficient information tools and single forms of credit in rural areas (Merrett and Gruidl, 2000; Constantinidis et al., 2006). Yunsong (2022) finds that female entrepreneurship faces more severe liquidity constraints than men, and female entrepreneurs are mainly financed by family-owned funds and less by external assistance. Qiang Guoling (2022) argues that female entrepreneurs often face financing constraints and the capital threshold required to start a business is difficult to cross.

As a comprehensive economic system relying on information technology, the Internet makes the entrepreneurship relying on the Internet media reflect unprecedented openness, borderlessness, and strong interaction (Reuber and Fischer, 2009; Etemad et al., 2010). Internet-based platforms of information, socialization, and transactions have overturned traditional interpersonal aggregation methods and rules, provided entrepreneurs with channels to disseminate entrepreneurial information and resources, lowered the cost and threshold of entrepreneurship, and broken through the limitations of time and space for entrepreneurial activities (Janson and Wrycza, 1999; Batjargal, 2007; Mack et al., 2017). Using data of NCTU and the World Bank, Guillén and Suárez (2001) found that the development of internet provided information, e-commerce, and chat services for entrepreneurship and investment, thus encouraging entrepreneurial activities and investment. Using longitudinal data from the China Household Tracking Survey (CFPS), Barnett et al. (2019) found that Internet use promoted entrepreneurship among rural households. Based on 2016 CFPS data, Zhou et al. (2020) found that Internet use made farmers more accessible in terms of information access and social interactions, thereby increasing farm household income levels and the probability of entrepreneurship. Therefore, for rural married women with children, Internet use is likely to be a breakthrough point for them to access entrepreneurial opportunities and start entrepreneurial activities. The popularity and use of Internet technologies not only break the traditional “information silo effect” and subjective gender bias, but also provide rural married women with children with more entrepreneurial opportunities and more flexible entrepreneurial schedules, and lower the entrepreneurial threshold and transaction costs, provide convenience for rural married women with children entrepreneurial activities, so that they are no longer subject to the constraints of time and space in the entrepreneurial process. Therefore, the following hypotheses are proposed in this paper.

*H1*: Internet use can significantly promote entrepreneurship among rural married women with children.

The transmission path of the impact of Internet use on the entrepreneurship of rural married women with children is multifaceted. In addition to directly reducing the information and transaction costs of the entrepreneurial activities of rural married women with children, internet use can also affect the entrepreneurship of rural married women with children through other ways. First, the Internet provides a more equitable entrepreneurial environment for rural married women with children entrepreneurs by empowering women economically and raising the concept of gender equality. Influenced by social attitudes and cultural environment, the entrepreneurial environment of rural married women with children often faces different degrees of gender discrimination and gender stereotypes, and people’s subconscious gender perceptions are more likely to accept that female entrepreneurs are less talented than men, and the bias against female entrepreneurs often makes them face gender discrimination (Harrison and Mason, 2007).

The Internet, on the other hand, provides more channels for women to seek power, improves gender equality perceptions through economic empowerment of women, and optimizes the entrepreneurial environment for rural married women with children. Second, the openness, growth and communication of the Internet broaden the access to information and knowledge for rural married women with children, and the flow of information provides more learning opportunities for rural women’s entrepreneurship and indirectly promotes their entrepreneurial behavior by improving rural women’s cognitive level (Nwankwo and Okeke, 2017; Nie et al., 2021). Johan and Cumming (2006) found that the Internet facilitated the dissemination of knowledge, and the spillover effect of knowledge motivated female entrepreneurship. Finally, Internet use helps to alleviate the financing constraints of rural married women with children. Entrepreneurial activities are inseparable from financial support, but due to the few financial institutions in rural areas, the low creditworthiness of rural married women with children, and the lack of effective collateral, the entrepreneurial activities of rural married women with children are generally faced with serious financial constraints. The combination of Internet information technology and inclusive finance has become an important part of overcoming financial exclusion and serving disadvantaged groups. Liu T. et al. (2021) found that the development of digital finance helped improve the rural entrepreneurial environment and equalized entrepreneurial opportunities. Luo et al. (2021) argued that digital inclusive finance provided a relatively convenient and feasible financing channel for low-income households that enabled them to break the financial barrier to start a business. Therefore, this paper proposes the following hypothesis.

*H2*: Internet has used gender perceptions, information transmission and financial support to stimulate entrepreneurial activity among rural married women with children.

The impact of Internet use on rural married women with children entrepreneurship is largely limited by macroeconomic level and micro-individual heterogeneity. Specifically, the information transmission effect and resource integration of Internet use are not only influenced by regional economic level, industrial structure and urbanization level, but the differences in education level and age of individual rural married women with children entrepreneurs also contribute to the impact of the Internet. From a macro perspective, regional economic level, industrial structure and urbanization level determine regional factor allocation and utilization efficiency. A region with a higher resource allocation efficiency has a higher Internet penetration rate and more opportunities for entrepreneurship and employment. Therefore, the entrepreneurial behavior of rural married women with children needs to consider the opportunity cost of entrepreneurship. When the opportunity cost of starting a business is high, rural married women with children will prioritize employment (Coleman, 2007). Only when the opportunity cost of starting a business is low, rural married women with children will consider starting a business. However, in areas with low resource allocation efficiency, the Internet penetration rate is relatively low, and the marginal utility of Internet information transmission effect and resource integration effect is higher, which is more favorable for rural married women with children entrepreneurs to use the information to integrate external factors and make up for their initial endowment shortcomings. Therefore, Internet use may have a stronger entrepreneurial effect on rural married women with children in less economically developed areas. Analyzed from a microscopic perspective, individual differences of rural female entrepreneurs influence the role of the Internet. For example, education level directly determines the cognitive level and learning ability of rural women, and rural women with higher education levels are more likely to benefit from the Internet for entrepreneurship, while the opposite is true for rural women with lower education levels. Accordingly, this paper proposes hypothesis 3.

*H3*: There are regional and individual differences in the impact of Internet use on rural female entrepreneurship.

## Research design

### Data sources

Considering the availability of data, the relevant data in this paper are mainly from the China General Social Survey CGSS 2017 and the China Statistical Yearbook. CGSS data cover 162 counties in 25 provinces (autonomous regions and municipalities), and the survey includes individuals and households. The China Statistical Yearbook provides the macro entrepreneurial environment at the provincial level. To ensure the reliability of the study, the data were processed as follows: first, rural women with inconsistent household registration and place of residence were excluded, considering the possible bias brought by the cross-district mobility of the rural population; second, rural women over 60 years old were excluded, and the sample was selected from rural women aged 18–60; third, school students and individuals who lost their labor force were excluded. Two thousand and twenty-eight samples were obtained. There were 148 entrepreneurial women among them, accounting for 7.2% of the total. There were 133 survival-oriented entrepreneurial women who made up 76.3% of all entrepreneurs, and 35 opportunity-oriented entrepreneurial women who made up 23.6%. Only 29.4% of the women in the sample used the Internet frequently or frequently, while 54.2% used it only occasionally or infrequently.

### Variable design

#### Explained variable

Rural female entrepreneurship (Entre). Referring to the study by Tan and Li (2022) and considering the availability of data, this paper is based on the questionnaire asking respondents “*What is your main type of work*?” Dummy variables were constructed. If interviewee answers “self-employed or I am the owner (or partner),” she is considered as the entrepreneurial sample with a value of 1, and if not, she is considered as the non-entrepreneurial sample with a value of 0. Considering the type of entrepreneurship, we refer to the study of Liu et al. (2019), the number of people under management is not 0 is defined as opportunity entrepreneurship and the number of people under management is 0 is defined as survival entrepreneurship.

#### Explanatory variable

Internet use (Inter). Referring to Zhou et al. (2020) study, this paper is based on a questionnaire that asked respondents, “In the past year, how much did you use the Internet?” Dummy variables were constructed, and the options were assigned 0–4 in the order of “never, rarely, sometimes, often, and very often.”

#### The control variables include three levels

First, the individual level, referring to the study of Wang et al. (2018), age (Age), age squared/100 (Age^2^), educational status (Edu), health (Health), and political affiliation (Policy) of rural women were used as control variables at the individual level. Educational attainment was expressed in years, and health was ranked from 0 to 4 in the order of very unhealthy, relatively unhealthy, generally healthy, relatively healthy, and very healthy. For political outlook, party members were assigned a value of 1 and non-party members were assigned a value of 1.

Second, at the household level, referring to the study by Ge et al. (2022), household income (Income), household size (Size), property status (House), and the presence of a vehicle (Car) were used as control variables at the household level for rural female entrepreneurship.

Third, at the macro-environmental level, drawing on Vander Zwan et al. (2012) study, the level of urbanization (Urban), industrial structure (Is), GDP *per capita* (PDP), and infrastructure development (Road) are used as control variables for the entrepreneurial environment. In this case, infrastructure development was replaced by road miles per square kilometer.

### Models

This paper focuses on the impact of Internet use on rural women’s entrepreneurship and considers whether rural women start a business as a binomial question, so a Probit model is chosen for the analysis. The Probit model is a dichotomous dependent variable model that assumes that the probability of an event follows a cumulative normal distribution function. Aiming to find the relationship between a set of characteristics describing an individual and the probability of that individual making a particular choice, we assume that each individual faces a choice between the two and that the choice depends on discriminable characteristics.A model of Internet usage choice was constructed, drawing on the model setting of Vander Zwan et al. (2012).

The dependent variable of the model is rural female entrepreneurship, and the probability of rural female entrepreneurship (i.e., *Y* = 1) is defined as *Y* = 1, and the probability of rural female entrepreneurship (i.e., *Y* = 1) is defined as *Y* = 0. The model between rural female entrepreneurship and internet usage is as follows.


(1) $$P\left(\mathrm{Entr}e_{i}=1\right)=\beta_{0}+\beta_{1}\mathrm{Inte}r_{i}+\beta_{2}\mathrm{Contro}l_{i}+\varepsilon_{i}$$

*β* is the coefficient to be estimated. Enter is a dummy variable for female entrepreneurship. Inter is the degree of Internet use. The control variables include individual, household, and macro entrepreneurial environment components.

To examine the transmission mechanism of Internet use in rural female entrepreneurship, drawing on Tan and Li (2022), four variables, gender perception, learning effect, financing situation, and social network, were introduced and tested as interaction terms with explanatory variables based on model 1. The model was designed as follows.

(2) $$\begin{array}{c} P\left(\mathrm{Entr}e_{i}=1\right)=\beta_{0}+\beta_{1}\mathrm{Inte}r_{i}+\beta_{2}Z_{i}+\beta_{3}\mathrm{Inte}{r_{i}}^{*}\;Z_{i}\\ \;+\beta_{4}\mathrm{Contro}l_{i}+\varepsilon_{i} \end{array}$$

*Z* denotes the conductive variables, which are gender perceptions, learning effects, and financing status, respectively. The interaction term is the multiplier of the conductive variable *Z* and the explanatory variable Internet use (Inter). Decentering the variables before empirical evidence to avoid potentially biased estimation effects due to multicollinearity.

### Descriptive statistics

Table 1 reports the descriptive statistics of the variables. As can be seen from Table 1, the proportion of rural married women with children is low, with less than 8% of the sample being female entrepreneurs, much lower than the 11.03% of male entrepreneurs. The mean value of the degree of Internet use is 1.28 and the standard deviation is 1.57, indicating that there are significant individual differences in the degree of Internet use among rural women. Regarding individual characteristics, the average age of rural women in the sample was 48.38 years old, the average education level was 6.52 years, and the health level was average. In terms of family characteristics, the average annual household income was 45,500 RMB, the family size was about 4 persons, the families all had one suit, and only 27.77% of the families owned a car. As for the entrepreneurial environment, the average urbanization level is 57.49%, the GDP *per capita* is 10971 yuan, and the mileage per square kilometer is 1.3km of road.

**Table 1:** Summary statistics.

Variables	Definition	Mean	SD	Min	Max
Rural married women with children entrepreneurship	Whether there is entrepreneurial activity (Yes = 1, No = 0)	0.0730	0.2602	0	1
Internet usage	Frequency of Internet use(0 = Never at all-4 = Highly frequent)	1.2835	1.5671	0	4
Age	The actual age of women (years old)	48.3826	10.5747	18	60
Age^2^/100	Square of women’s actual age	24.5265	9.8040	3.24	40.96
Education level	Education background (years)	6.5197	4.0723	0	18
Health level	State of health (0 = not healthy at all-4 = very healthy)	2.3082	1.1309	0	4
Political appearance	Member of the Communist Party of China (Yes = 1, No = 0)	0.0276	0.1639	0	1
Family income	Total income of the household (yuan/Year)	45487.76	100671.3	0	3,000,000
Real estate	Property owned by a family (set)	1.0971	0.5385	0	11
Cars	Availability of a car (yes = 1, no = 0)	0.2342	0.4236	0	1
Family size	Number of family members	3.3185	2.6324	1	9
Urbanization	Urban population as a proportion of total population (%)	1.0706	0.4610	46.02	87.70
Infrastructure development	Road miles per square kilometre	1.3041	0.5544	0.12	2.10
Industrial structure index	Quantitative comparison of output by industry	1.30	0.55	0.94	4.89
Population density	Number of people per square kilometre	399.68	448.3	5.06	3838.10
GDP *per capita*	Gross National Product *per capita* (million yuan)	1.0971	0.5385	2.91	13.62

To ensure that there was no multicollinearity between the variables, a Person test was done, as shown in Table 2, which showed a maximum value of 0.66, below 0.8. The variance inflation factor test showed that the maximum value of VIF was 1.6, which is much less than 10. Therefore, there is no multicollinearity.

**Table 2:** Correlation test.

Variables	WE	IMU	AGE	AGE2	EDU	HL	PA	FI	RE	CAR	FZ	URB	PD	GDP	ID	ISI
WE	1															
IMU	0.1489	1														
AGE	0.1186	0.5975	1													
AGE2	−0.1272	−0.5907	0.5928	1												
EDU	0.1225	0.4969	−0.3911	−0.3943	1											
HL	0.0996	0.295	−0.3293	−0.3255	0.2827	1										
PA	0.0337	0.1366	−0.0801	−0.0766	0.1936	0.0845	1									
FI	0.1265	0.326	−0.2285	−0.2422	0.2925	0.2998	−0.0435	1								
RE	0.0374	0.0498	0.0111	0.007	0.0771	0.0521	0.0646	0.1617	1							
CAR	0.0821	0.2633	−0.1455	−0.1429	0.2383	0.2035	0.0844	0.3294	0.2073	1						
FZ	0.0042	0.0813	−0.1299	−0.1242	0.0134	0.0379	−0.0055	0.0824	0.0721	0.1007	1					
URB	0.0085	0.1749	0.0206	0.0235	0.1533	0.1363	0.0312	0.2887	0.0747	0.1634	−0.0109	1				
PD	0.008	0.0899	0.011	0.0138	0.0822	0.1583	0.0174	0.1899	0.0855	0.1347	0.0276	0.514	1			
GDP	0.0258	0.1193	0.0201	0.0235	0.0968	0.1694	0.0457	0.29	0.0753	0.1475	0.0002	0.6597	0.6511	1		
ID	0.0117	0.0301	0.0202	0.0251	0.0248	0.1415	0.0123	0.1429	0.0682	0.092	0.0196	0.3604	0.6617	0.5816	1	
ISI	−0.0213	0.117	0.0283	0.0271	0.1452	0.0138	0.0321	0.082	0.0469	0.1332	−0.0387	0.4826	0.1162	0.3052	−0.0825	1

## Empirical analysis

### Baseline regression

Table 3 reports the Probit regression results. Model (1) and (2) show the estimation results without and with control variables, respectively, while Model (3) and (4) show the estimation results for survival entrepreneurship and opportunity entrepreneurship, respectively. Model (1) shows that the marginal effect of Internet use on rural married women with children entrepreneurship is 0.169 and is significant at the 1% level, indicating that Internet use increases the rural married women with children entrepreneurship rate. Model (2) shows that the marginal utility of Internet use on rural married women with children entrepreneurship decreases to 0.0788 after adding control variables and is significant at the 5% level. This verifies hypothesis 1 of this paper that Internet use promotes entrepreneurship among rural married women with children. Model (3) shows that the marginal effect of Internet use on survival-oriented entrepreneurship among rural married women with children is 0.062 and significant at the 10% level. Model (4) shows that the marginal effect of Internet use on rural married women with children opportunity-based entrepreneurship is 0.0045, but it does not pass the significance test. This indicates that there is a difference in the effect of Internet use on different types of entrepreneurship among rural married women with children, and the effect of Internet use on survival entrepreneurship among rural married women with children is greater than that of opportunity entrepreneurship.

**Table 3:** Benchmark regression results.

Variables	(1)	(2)	(3)	(4)
	Entrepreneurship	Entrepreneurship	Survival	Opportunistic
Internet usage	0.1692^***^	0.0788^**^	0.0620^*^	0.0045
	(0.0251)	(0.0384)	(0.0407)	(0.0371)
Age		0.157^***^	0.164^***^	−0.0651
		(0.0407)	(0.0449)	(0.171)
Age^2^/100		−0.1822^***^	−0.1894^***^	0.0718
		(0.0461)	(0.0502)	(0.199)
Education level		0.0314^**^	0.0321^*^	0.2584^***^
		(0.0153)	(0.0173)	(0.0757)
Health level		0.0626	0.0997^*^	−0.287
		(0.0484)	(0.0530)	(0.2217)
Political appearance		−0.0569	0.0409	−0.497
		(0.2513)	(0.2792)	(0.5726)
Family income		0.1255^**^	0.0679	1.1388^**^
		(0.0601)	(0.0581)	(0.4632)
Real estate		0.0810	0.1112	−0.1244
		(0.0783)	(0.0826)	(0.3724)
Cars		0.0914	0.0565	−0.7091
		(0.112)	(0.126)	(0.485)
Family size		−0.00842	−0.0376	0.5073^***^
		(0.0201)	(0.0390)	(0.1461)
Urbanization		−0.0123	−0.0055	−0.0545
		(0.0130)	(0.0139)	(0.0554)
Population density		−0.0011^*^	−0.0008	−0.0061^*^
		(0.0005)	(0.0005)	(0.0034)
GDP *per capita*		0.5412	0.4554	0.6137
		(0.3373)	(0.3587)	(1.3351)
Infrastructure development		0.0571	0.0948	2.4474^*^
		(0.2381)	(0.2523)	(1.2692)
Industrial structure index		0.0571	0.0656	0.8920^*^
		(0.1172)	(0.1211)	(0.4901)
Constant	−1.724^***^	−12.33^***^	−11.22^***^	−16.15
	(0.0614)	(3.3634)	(3.5827)	(13.911)
*N*	2028	1807	1722	85
Pseudo *R*^2^	0.0403	0.0958	0.0806	0.4160

^***^, ^**^, and ^*^ indicate that the estimation results are significant at the 1, 5, and 10% levels, respectively. The robust standard errors are in parentheses. Model (1) is the estimated result for the inclusion of control variables. Model (2) is the estimated result for the inclusion of control variables; Model (3) is the estimated result for survival entrepreneurship, and Model (4) is the estimated result for opportunity entrepreneurship.

Such results may be related to female entrepreneurial motivation. Birley (1987) argued that female entrepreneurial motivations could be divided into push and pull motivations. Push motivations are caused by insufficient income, unemployment and underemployment. Pull motivations are caused by the search for independence, personal interest in business and judgment of expected market opportunities. Kalemci Tuzun and Araz Takay (2017) further found that the motivation of female entrepreneurs in rural areas was dominated by the push effect, while the motivation of urban female entrepreneurs was dominated by the pull effect. Thus, for rural married women with children, insufficient income, unemployment and underemployment drive their choice of survival entrepreneurship.

In addition, the regression results of the control variables show that the age of rural married women with children entrepreneurs is significantly and positively related to the marginal effect of their entrepreneurship, and the age squared is significantly and negatively related to the marginal effect of their entrepreneurship, therefore, the age of rural married women with children and entrepreneurial activity are inverted “U” shaped. Educational level and household income were significantly and positively associated with entrepreneurship among rural married women with children. There is no significant effect of health, political affiliation, family size, property, urbanization, industrial structure, GDP *per capita*, and infrastructure on rural women’s entrepreneurship.

### Robustness tests

To ensure the reliability of the baseline regression results, the following robustness test is adopted in this paper, as shown in Table 4.

**Table 4:** Robustness tests.

Variables	(1)	(2)	(3)
	OLS	Excluding variables	Tool variables
Internet usage	0.0133^**^	0.0828^**^	0.2902^***^
	(0.0058)	(0.0410)	(0.0407)
Age	0.0141^***^	0.1554^***^	0.1656^***^
	(0.0042)	(0.0437)	(0.0504)
Age^2^/100	−0.0158^***^	−0.1817^***^	−0.1758^***^
	(0.0044)	(0.0496)	(0.0503)
Education level	0.0034^**^	0.0370^**^	0.0102
	(0.0016)	(0.0166)	(0.0246)
Health level	0.0071	0.0473	0.0432
	(0.0059)	(0.0517)	(0.0502)
Political appearance	0.0154	−0.0907	−0.0118
	(0.0464)	(0.264)5	(0.0344)
Family income	0.0125^**^	0.1434^**^	0.0950
	(0.0050)	(0.0633)	(0.0670)
Real estate	0.0103	0.0782	0.0795
	(0.0130)	(0.0828)	(0.0807)
Cars	0.0195	0.0743	0.0697
	(0.0177)	(0.1183)	(0.1284)
Family size	−0.0006	−0.0011	0.0189
	(0.0012)	(0.0116)	(0.0351)
Urbanization	−0.0023	−0.0201	−0.0259^*^
	(0.0015)	(0.0131)	(0.0150)
Population density	0.0606	0.9081^**^	0.4964
	(0.0443)	(0.3541)	(0.3693)
GDP *per capita*	−0.0108	0.0549	−0.0278
	(0.0307)	(0.3103)	(0.237)
Infrastructure development	−0.0132	0.8602^**^	−0.0685
	(0.0121)	(0.3391)	(0.1222)
Industrial structure index		−0.0004	−0.0685
		(0.0007)	(0.1218)
Constant	−0.8947^**^	−16.5710^***^	−10.5303^***^
	(0.4211)	(3.7096)	(3.6053)
*N*	2,028	1,569	2,028
Pseudo *R*^2^	0.0415	0.1099	0.4796
Phase I *F*-value			112.64
Wald endogeneity test			0.82
Wald chin^2^			62.13^***^

^***^, ^**^, and ^*^ indicate that the estimation results are significant at the 1, 5, and 10% levels, respectively. The robust standard errors are in parentheses.

#### Replacement of regression methods

In terms of the characteristics of the data itself, although the dependent variable of rural married women with children starting a business is discrete, the control variables measuring the entrepreneurial environment are all non-discrete variables. Considering the non-discrete characteristics of the control variables data, this paper further estimated with the OLS model based on the Probit model. Column (1) in Table 4 shows that the estimated coefficient of Internet use is 0.013 and passes the significance test, indicating that Internet use promotes rural female entrepreneurship. This is generally consistent with the baseline regression results.

#### Excluding special samples

Considering the outliers in the sample, the data are re-estimated by excluding the abnormal data as follows: First, the control of regions. The municipalities are important provincial-level administrative regions in China, which are pivotal in terms of economic strength and influence, while the autonomous regions are the opposite; therefore, the samples from Shanghai, Beijing, Tianjin, Chongqing, Inner Mongolia, and Guangxi are excluded; second, the control of age, to ensure that rural women still have entrepreneurial vitality and have not withdrawn from the market, women who are older than 55 years old are excluded from the sample. Column (2) shows that Internet use is significantly and positively related to rural married women with children entrepreneurship, indicating that Internet use has a significant positive contribution to rural married women with children entrepreneurship. It indicates that the findings of this paper remain robust.

#### Instrumental variables approach

Given the possibility of reverse causality between Internet use and rural pregnant women’s entrepreneurship, leading to biased model estimates generate endogeneity problems. To weaken this situation, this paper refers to Luo et al. (2021), using “How many devices currently have Internet access” as an instrumental variable to estimate the baseline regression. Wald’s endogeneity test shows that Internet use rejects the original hypothesis of homogeneity. From the empirical results, the marginal effect of Internet use on rural female entrepreneurship is significantly and positively correlated, indicating that Internet use has a positive contribution to rural pregnant female entrepreneurship. This is consistent with the regression results in the previous paper, indicating the robustness of the estimation results in this paper.

### Heterogeneity analysis

#### Different education levels

Educational attainment directly determines the cognitive ability of individual rural women and also plays a role in their learning ability and financial literacy. Davidsson and Honig (2003) argue that access to education is a process of human capital accumulation and that farmers with high levels of academic education are more likely to start their businesses. Considering the importance of education in the process of rural entrepreneurship, this paper further explores the possible effects of Internet use on entrepreneurship among rural pregnant women with different levels of education. Based on the education level of rural pregnant women, their education level was divided into two groups according to their average education level: low education level and high education level. Table 5 shows that Internet usage has no significant effect on entrepreneurship of rural less educated women, while it has a significant impact on rural women graduated from junior high school and above.

**Table 5:** Different education levels.

Variables	(1)	(2)
	High education	Low education
Internet usage	0.1544^***^	0.0214
	(0.0571)	(0.0513)
Age	0.2435^***^	0.1347^***^
	(0.0816)	(0.0520)
Age^2^/100	−0.2654^***^	−0.1608^***^
	(0.0883)	(0.0614)
Education level	−0.0035	−0.0308
	(0.0311)	(0.0315)
Health level	0.1518**	−0.0033
	(0.0677)	(0.0666)
Political appearance	0.6124	−0.0199
	(0.6579)	(0.2691)
Family income	0.1163	0.1567^*^
	(0.0978)	(0.0884)
Real Estate	0.1482	−0.0171
	(0.1137)	(0.1095)
Cars	−0.2453	0.2677^*^
	(0.2295)	(0.1379)
Family size	−0.0531	0.0215
	(0.0533)	(0.0456)
Urbanization	−0.0109	−0.0108
	(0.0208)	(0.0166)
Population density	−0.1363	0.4630^**^
	(0.2284)	(0.2021)
GDP *per capita*	0.1560	0.7261^*^
	(0.510)	(0.4252)
Infrastructure development	0.4701	−0.2314
	(0.3877)	(0.2996)
Industrial structure index	0.2602	0.0607
	(0.1759)	(0.1418)
Constant	−9.7223^*^	−14.5051^***^
	(5.1348)	(4.2523)
*N*	994	813
Pseudo *R*^2^	0.1172	0.0695

^***^, ^**^, and ^*^ indicate that the estimation results are significant at the 1, 5, and 10% levels, respectively. The robust standard errors are in parentheses.

#### Different entrepreneurial environments

The entrepreneurial environment determines the efficiency of factor allocation and utilization and reflects the external dependence of rural female entrepreneurship. Minniti (2010) argues for a U-shaped relationship between GDP *per capita* and female entrepreneurship. Considering the impact of the entrepreneurial environment on rural women’s entrepreneurial decisions, this paper further explores the possible impact of Internet use on rural pregnant women’s entrepreneurship in different entrepreneurial environments. According to different regional economic development levels, regions with higher-than-average GDP *per capita* are defined as developed regions’ entrepreneurial environments, and regions with lower-than-average GDP *per capita* are defined as less developed regions’ entrepreneurial environments. Table 6 illustrates that Internet usage has a significant promotive effect on rural pregnant women’s entrepreneurship, but in less developed areas, the marginal effect of rural pregnant women’s entrepreneurship is higher than it is in developed regions. This also verifies hypothesis 2, that Internet use has a stronger effect on entrepreneurship among rural married women with children in less economically developed regions.

**Table 6:** Different entrepreneurial environments.

Variables	(1)	(2)
	Less developed areas	Developed regions
Internet usage	0.0971^**^	0.0651^*^
	(0.0476)	(0.0564)
Age	0.1790^***^	0.1121
	(0.0493)	(0.0713)
Age^2^/100	−0.2104^***^	−0.1252
	(0.0558)	(0.0796)
Education level	0.0248	0.0575**
	(0.0201)	(0.0249)
Health level	0.0447	0.0961
	(0.0584)	(0.0880)
Political appearance	0.2002	−0.8183^*^
	(0.3186)	(0.4531)
Family income	0.1257^*^	0.1412
	(0.0738)	(0.1136)
Real estate	0.1337	−0.0270
	(0.0935)	(0.1455)
Cars	0.2482*	−0.2427
	(0.1381)	(0.1917)
Family size	−0.0267	0.0356
	(0.0422)	(0.0589)
Urbanization	−0.0239^*^	−0.0071
	(0.0145)	(0.0381)
Population density	0.0001	−0.0038^**^
	(0.0013)	(0.0018)
GDP *per capita*	2.4074^***^	0.5817
	(0.7582)	(0.7653)
Infrastructure development	−0.4955	0.2887
	(0.5561)	(0.4449)
Industrial structure index	0.6143	0.03847
	(0.4646)	(0.246)
Constant	−31.6845^***^	−22.1627^**^
	(8.0183)	(8.6692)
*N*	1,147	660
Pseudo *R*^2^	0.1338	0.1376

^***^, ^**^, and ^*^ indicate that the estimation results are significant at the 1, 5, and 10% levels, respectively. The robust standard errors are in parentheses.

## Exploration of mechanisms

### Gender perspective and female entrepreneurship

Gender perceptions have a significant impact on rural entrepreneurial activities. Studies show that the more pronounced the trend of gender equality, the more it contributes to female employment and entrepreneurship. Franzke et al. (2022) concluded that equal gender perceptions have a significant positive impact on rural women’s educational attainment, labor participation, and career development. The Internet empowers women by spreading equal gender perceptions and provides more convenience for rural female entrepreneurs. Considering the transmission effect of gender perceptions, this paper constructs dummy variables based on the questionnaire that asks respondents “*Men put their careers first and women put their families first*,” in which the value of fully agree is 0, relatively agree is 1, indifferent is 2, relatively disagree is 3, and completely disagree is 4, and constructs the interaction term between the core independent variable Internet use and The interaction term between the core independent variable Internet use and gender perception. Table 7 model (1) reveals that Internet use, gender perceptions and interaction term are significantly and positively related to rural pregnant women’s entrepreneurship, suggesting that the improvement of rural women’s gender perceptions promotes the impact of Internet use on rural women’s entrepreneurship.

**Table 7:** Analysis of conduction mechanisms.

Variables	(1)	(2)	(3)
Internet usage	0.1457^**^	0.0523^*^	0.0670^*^
	(0.0584)	(0.0279)	(0.0393)
Gender perspective	0.1932^***^		
	(0.0626)		
Internet * gender	0.0416^*^		
	(0.0240)		
Learning effect		0.1657^*^	
		(0.0835)	
Internet * learning		0.0566^**^	
		(0.0256)	
Financial empowerment			0.1697^*^
			(0.0892)
Internet * financial			0.207^*^
			(0.116)
Age	0.1594^***^	0.1674^***^	0.1549^***^
	(0.0436)	(0.0416)	(0.0408)
Age^2^/100	−0.1835^***^	−0.1931^***^	−0.1797^***^
	(0.0486)	(0.0469)	(0.0462)
Education level	0.03085^**^	0.0323^**^	0.0303^**^
	(0.0154)	(0.0154)	(0.0154)
Health level	0.0623	0.0651	0.0647
	(0.0494)	(0.0481)	(0.0487)
Political appearance	−0.0950	−0.0908	−0.1083
	(0.246)	(0.258)	(0.263)
Family income	0.1236^**^	0.1265^**^	0.1229^**^
	(0.0601)	(0.0602)	(0.0603)
Real estate	0.0953	0.0832	0.0731
	(0.0793)	(0.0786)	(0.0800)
Cars	0.0865	0.0920	0.0908
	(0.1152)	(0.1127)	(0.1124)
Family size	−0.0055	−0.0102	−0.0078
	(0.0236)	(0.0229)	(0.0195)
Urbanization	−0.0096	−0.0116	−0.0134
	(0.0139)	(0.0130)	(0.0130)
Population density	−0.0009^*^	−0.0010^*^	−0.0010^*^
	(0.0005)	(0.0005)	(0.0005)
GDP *per capita*	0.4683	0.5221	0.5691^*^
	(0.3591)	(0.3378)	(0.3382)
Infrastructure development	0.0536	0.0769	0.0538
	(0.2328)	(0.2397)	(0.2383)
Industrial structure index	0.0393	0.0473	0.0466
	(0.1314)	(0.1186)	(0.1123)
Constant	−12.0236^***^	−12.3176^***^	−12.4652^***^
	(3.5594)	(3.3692)	(3.3607)
*N*	1,807	1,807	1,807
Pseudo *R*^2^	0.1072	0.1007	0.1006

^***^, ^**^, and ^*^ indicate that the estimation results are significant at the 1, 5, and 10% levels, respectively. The robust standard errors are in parentheses.

### Learning effect and female entrepreneurship

Internet use provides more learning opportunities for pregnant rural women, which helps to improves women’s human capital and thus further improve women’s entrepreneurial skills. Liu et al. (2016) analyzed the mediating role of entrepreneurial learning from the perspective of Internet embedding, and found that the more individuals use the Internet, the easier it is to obtain more information and promote resource acquisition through the learning effect. The Internet delivers information such as news, videos, and digital resources to entrepreneurs in the form of online media embedding, which lowers the threshold of learning and resource acquisition for entrepreneurs. Entrepreneurs, in turn, improve their entrepreneurial skills through learning, training, etc. Considering the learning utility of the Internet, this paper uses the questionnaire question “*How much time do you spend each day reading information and articles on your computer or through various mobile applications such as WeChat and Weibo*” as a proxy variable for learning utility and constructs an interaction term between the core independent variable Internet use and learning utility. Table 7 model (2) shows that Internet use, learning utility and interaction term are significantly and positively related to rural pregnant women’s entrepreneurship, indicating that the learning utility of Internet use contributed to the influence of Internet use on rural women’s entrepreneurship.

### Financial empowerment and female entrepreneurship

Digital inclusive finance supported by Internet digital technology makes up for the shortcomings of traditional financial services through scenarios, data, information, and innovation, and gives full play to the advantages of “wide coverage, low cost and fast,” thus effectively alleviating the financing constraints of disadvantaged groups such as poor farmers, new agricultural business entities and rural micro and small enterprises (Aisaiti et al., 2019; Luo and Zeng, 2020). Liu et al. (2019) found that the development of digital technology helped to improve the rural entrepreneurial environment and equalize entrepreneurial opportunities. Therefore, for rural pregnant entrepreneurial women, the development of digital technologies on the Internet is likely to be a breakthrough point for them to address their financing constraints. Considering the financial empowerment of the Internet, this paper constructs dummy variables based on the questionnaire that asks respondents “*If you need a large amount of money, who or which institution would you ask for help*,” where family or close friends and other people are assigned a value of 0 and commercial institutions are assigned a value of 1. The core independent variables of Internet use and financial empowerment are also constructed. The interaction term between the core independent variables of Internet use and financial empowerment was constructed. From Table 7 model (3), we can see that Internet use, financial empowerment and interaction term are significantly and positively related to rural pregnant women’s entrepreneurship, indicating that financial empowerment of Internet use facilitates the effect of Internet use on rural women’s entrepreneurship.

## Discussion

Employing the data conveyed by *China Social Survey* to link rural married women’s entrepreneurship with Internet application, we empirically demonstrated that the widespread use of information technology indeed does enhance rural married pregnant women’s entrepreneurship; this proves that these women make enormous use of the Internet for the purpose of accessing highly valuable and sought for entrepreneurial opportunities.

However, these findings are not consistent with the view of some scholars who argue the existence of a ‘digital divide’ in the geospatial and social structure of InfoTech usage that hinders rural married-pregnant women’s entrepreneurial activities (Omotoso et al., 2020; Acilar and Sæbø, 2021). They point out that, firstly: this ‘digital divide’ in the social structure is attributed to individual characteristics along with the parallel communication systems created by the network society. In turn, this network society provides low-cost high-speed information resources to high-income, highly educated, and highly accessible people whilst leaving low-income, low-education, and low participants in the public system out in the cold openly ignored and excluded by the Internet exclusion standards (Antonio and Tuffley, 2014). Sad, but true, the low levels of education, weak capacity to accept new technologies, and high costs of Internet connectivity prevent rural mothers from making effective use of the Internet for entrepreneurship (Puja, 2019).

Secondly, the geospatial ‘digital divide’ is a result of the economic base, where technological infrastructure, wealth and educational resources are unequally distributed between rural and urban areas leading to disequilibrium in these regions’ usage of information technology (Dutton et al., 2004). Poor economic development and severe resource constraints in rural areas prevent rural entrepreneurs, specifically, children-raising women, with family responsibilities added, from maximizing their usage of the benefits the Internet can offer for starting their own businesses (Erogul et al., 2019). A possible explanation for the discrepancy in the findings is that extant studies on the matter generally ignore the initiative and risk-tolerance of such women addressed above (Shepherd and Patzelt, 2011; Si et al., 2022b). Adding to this, they tend to shy away from mentioning the inclusiveness of Internet technology as well (Balagopal, 2020).

On the positive side, some scholars suggest that if the ‘digital divide’ is removed, rural women would proactively use the Internet under risk-controlled conditions, and the utility of information learning from the Internet will help them escape the gender digital divide and better facilitate their search for entrepreneurial opportunities (Ramos and Prieto, 2014). Liu P. et al. (2021); Kerras et al. (2020). In fact, many other scholars have validated these findings.

According to our study, it was discovered that while Internet application enhances the level of entrepreneurship among rural married and/or pregnant women, there is considerable heterogeneity, highlighted by two factors – level of education, and type of entrepreneurship. This discovery also confirms the findings of Klasen and Lamanna (2009); Kalemci Tuzun and Araz Takay (2017), and others. These experts affirm that the level of education determined rural women’s ability to learn; and that the higher the level of education, the higher their ability to learn and the higher their likelihood of starting a business. Furthermore, when considering family responsibilities and balancing family income, family-caring women in rural areas lean more towards utilizing Internet-provided information for running subsistence businesses requiring low capital investment, relatively free time and little or no labour at all. In addition, we found that the age at which these women embark on starting an online business is in an inverted ‘U’ shape (the inflection point of the inverted “U” shape being at 44.83 years). This may be explained by the fact that women’s ability to learn and adapt to new technologies depletes with age.

## Research limitations

Although we have made some innovative contributions to the study of the perspectives and transmission mechanisms of female entrepreneurship, there are still some shortcomings that may provide new avenues of research for other scholars and researchers.

Firstly, given the limited data we had at our disposal, we only analyzed rural married and/or pregnant entrepreneurial behavior; we did not discuss the entrepreneurial performance or scale effects of these women. Secondly, the quantification of internet use in the academy still lacks protocols, thus we only measured this variable in terms of whether or not they employ the Internet in their businesses; we had no means to further analyse the heterogeneity of Internet usage. Finally, while it is affirmed by various scholars that female entrepreneurship – an emerging force for economic growth – will remain a hot topic in the near future. However, in academia, research on this subject matter still greatly lacks in sound theoretical analysis (Alderete, 2017). Hence, there is a need to expand the theoretical perspective, scope and research methods on female entrepreneurship.

## Conclusion and recommendations

In the context of digital economic development and rural economic transformation, it is important to explore the intricate correlations between Internet use and rural married women with children entrepreneurship to achieve rural revitalization and promote inclusive economic development and social progress.

This paper theoretically and empirically analyzes the impact and transmission mechanism of Information Technology (InfoTech) application on rural women’s entrepreneurship by applying the *China Integrated Social Survey*. In the benchmark regression, it is found that for every 1% increase in Internet use results in the probability of rural women’s entrepreneurship rise by 7.88%. Another method used for this paper’s findings was that the robustness of the findings was tested by the instrumental variable method, replacement regression, and exclusion of special values. We discovered that heterogeneity analysis demonstrated that usage of the Internet had no significant effect on rural women with low education level entrepreneurship. On the flipside, it had a significant appealing effect on rural women with junior high school education and above. Furthermore, making use of the Internet had a tremendously greater promotional effect on rural pregnant women’s entrepreneurship; but showing that it had a higher marginal effect on rural pregnant women’s entrepreneurship in less developed areas than in developed areas.

In regards to the mechanism analysis, it found that rural women’s gender perceptions were elevated to promote the impact of the Internet on rural female entrepreneurship. The increased learning utility and the financial empowerment prompted by Internet use resulted in an increase in the rate of rural women’s entrepreneurship. Hence, based on the above findings, this paper makes the following recommendations:

- Strengthen the construction of digital villages by improving the environment for the development of inclusive finance such as online payment, mobile payment, and online credit; and, of course, raise the level of rural women’s awareness.
- Promote the concept of gender equality, and promote the change of gender concept of rural residents in multiple channels and ways.

Continuously improve the education system and training methodology for rural women through strong support for the education of women and improvement of the education concept for farmers whilst continuously strengthening the entrepreneurship and employment training of rural women.

## Data availability statement

Publicly available datasets were analyzed in this study. This data can be found at: http://cgss.ruc.edu.cn/info/1014/1019.htm (CGSS2017).

## Author contributions

KS, LZ and KW: acquisition, analysis and interpretation of data; drafting. RM and JK: conception of the work; revising. All authors contributed to the article and approved the submitted version.

## Conflict of interest

The authors declare that the research was conducted in the absence of any commercial or financial relationships that could be construed as a potential conflict of interest.

## Publisher’s note

All claims expressed in this article are solely those of the authors and do not necessarily represent those of their affiliated organizations, or those of the publisher, the editors and the reviewers. Any product that may be evaluated in this article, or claim that may be made by its manufacturer, is not guaranteed or endorsed by the publisher.
